# Beta-Glucans Improve Growth, Viability and Colonization of Probiotic Microorganisms

**DOI:** 10.3390/ijms13056026

**Published:** 2012-05-18

**Authors:** Pasquale Russo, Paloma López, Vittorio Capozzi, Pilar Fernández de Palencia, María Teresa Dueñas, Giuseppe Spano, Daniela Fiocco

**Affiliations:** 1 Promis Biotech, Faculty of Agriculture, University of Foggia, Via Napoli 25, 71122 Foggia, Italy; E-Mails: p.russo@unifg.it (P.R.); vittorio.capozzi@gmail.com (V.C.); 2 Department of Food Sciences, Faculty of Agriculture, University of Foggia, Via Napoli 25, 71122 Foggia, Italy; 3 Biology Research Center, Department of Molecular Microbiology and Infection Biology, 28040 Madrid, Spain; E-Mails: plg@cib.csic.es (P.L.); pfpalencia@cib.csic.es (P.F.P.); 4 Department of Applied Chemistry, University of Basque Country (UPV/EHU), Paseo Manuel de Lardizábal 3, 20018 Donostia, Spain; E-Mail: mariateresa.duenas@ehu.es; 5 Department of Biomedical Science, Faculty of Medicine and Surgery, University of Foggia, Via Pinto, 1, 71122 Foggia, Italy; E-Mail: d.fiocco@unifg.it

**Keywords:** β-glucans, probiotics, prebiotics, *Lactobacillus plantarum*

## Abstract

Probiotics, prebiotics and synbiotics are frequently-used components for the elaboration of functional food. Currently, most of the commercialized probiotics are limited to a few strains of the genera *Bifidobacteria*, *Lactobacillus* and *Streptococcus*, most of which produce exopolysaccharides (EPS). This suggests that the beneficial properties of these microorganisms may be related to the biological activities of these biopolymers. In this work we report that a 2-substituted-(1,3)-β-d-glucan of non-dairy bacterial origin has a prebiotic effect on three probiotic strains. Moreover, the presence of this β-d-glucan potentiates *in vitro* adhesion of the probiotic *Lactobacillus plantarum* WCFS1 to human intestinal epithelial cells.

## 1. Introduction

Probiotics, prebiotics and synbiotics (a mixture of pro- and prebiotics) are today the most frequent components used for the elaboration of functional foods [1]. Probiotics are viable microorganisms able to reach the intestine in an active state and thereby exert positive health effects [2]. A prebiotic is a selectively fermented ingredient that promotes specific changes in the composition and/or activity of the gastrointestinal microbiota which, in turn, confers benefits on host well-being and health [3–5]. Non-digestible oligosaccharides (NDO) fulfill all the criteria for classification as prebiotics. Specifically, the bifidogenic NDO inulin, its hydrolysis product oligofructose, (trans)-galactooligosaccharides and lactulose are the prototype of prebiotic saccharides. Moreover, polysaccharides themselves, or as a source of NDO, are considered potential prebiotics. Bacteria can synthesize, and secrete through the cell wall, polysaccharide layers on their surface, which, together with a few glycoproteins, constitute the glycocalyx. These exocellular polymers comprise the capsular polysaccharides, which form a cohesive layer or capsule covalently linked to the cell surface, and the exopolysaccharides (EPS), which form a slime layer loosely attached to the cell surface or are secreted as free biopolymers into the environment.

Some EPS are important virulence determinants of pathogens, whilst others provide the bases for biotechnological applications [6]. Some EPS have been approved as food additives by the US Food and Drug Administration: the xanthan produced by *Xanthomonas campestris*, the gellan gum produced by *Sphingomonas paucimobilis* and the curdlan produced by *Agrobacterium*.

Lactic acid bacteria (LAB) are able to produce hetero- or homo-exopolysaccharides [7] and these EPS play an important role in the rheology, texture, and mouthfeel of fermented milks and other fermented products. In addition, health benefits have been claimed for EPS from LAB because of their putative antitumoral, immunostimulatory and blood cholesterol lowering activities [8].

Among EPS-producing LAB, *Pediococcus parvulus* 2.6, isolated from a ropy cider, produces a (1→3)-β-d-glucan homopolysaccharide with 50% of substitutions at positions *O*-2 by side chains composed of a single residue of glucose (2-substituted (1,3)-β-d-glucan) and with a molecular mass higher than 10^6^ Da [9]. Analysis of the rheological properties of this β-glucan showed that it has potential utility as a biothickener [10]. The β-glucan produced by *P. parvulus* 2.6 belongs to a group of linear and branched polysaccharides, the (1,3)-β-d-glucans, which are produced by several bacteria such as EPS, and are also found in fungi, cereals, and algae [11]. (1,3)-β-glucans are considered as biological response modifiers, and are attracting attention from the pharmaceutical and functional food industries because of their beneficial effects on human and animal health. Their biological effects are influenced by their degree of branching, molecular mass and tertiary structure [12]. Production of β-glucans confers to *Pediococcus* and *Lactobacillus* strains either an increased adherence to Caco-2 human enterocytes or a macrophage-immunomodulatory capacity [13,14]. Moreover, synthesis of the *P. parvulus* 2.6 β-glucan confers to *Lactobacillus paracasei* NFBC 338 higher survival during gastrointestinal passage or technological process [15]. Finally, the production of oat-based products, yogurt and various beverages using the 2-substituted (1,3)-β-d-glucan-producing LAB, significantly increased the techno-functional properties of these strains [16].

Human consumption of a ropy, oat-based product, co-fermented with the *P. parvulus* 2.6 strain, reduced serum cholesterol levels in humans, over and above the effect previously verified for the 4-substituted (1,3)-β-d-glucan from oat [17], suggesting a potential application of (1,3)-β-glucan-producing LAB for the elaboration of functional food.

Prebiotic effects of several EPS produced by LAB have been also reported [18], and availability of prebiotics specifically targeted at the probiotics strains could enable the development of symbiotic versions with enhanced survival in the gut [19].

In order to extend our knowledge on the prebiotic effect of EPS from microbial origin, we evaluated the effect of purified 2-substituted (1,3)-β-d-glucan on the growth of three probiotic strains, including a recombinant *Lactobacillus plantarum* strain that over-expresses a β-glycosidase enzyme. The influence of EPS on the capability of *L. plantarum* to adhere to human intestinal cells was also investigated. The results reported in this paper provide additional information on the positive effect of EPS as prebiotic substrates.

## 2. Results and Discussion

### 2.1. Overexpression of a β-Glycosidase Gene in *L. plantarum* WCFS1

**Figure 1:**
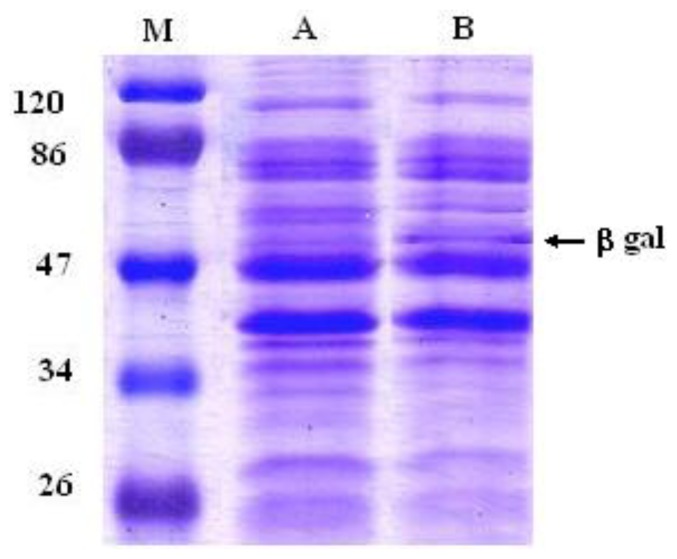
Sodium dodecylsulphate-polyacrylamide gel electrophoresis (SDS-PAGE) profiles of total protein extracts from *L. plantarum* WCFS1 harboring pGIZ906 (lane A) or the recombinant PldhL-bgl-pGIZ906 (strain WCFS1β-gal, lane B). The molecular size was determined by comparison with a protein ladder (M). M: prestained SDS-PAGE standards, low range. Molecular mass of the marker bands is indicated in kDa.

Since EPS are macromolecules that cannot cross the cell membrane through the common transport systems, they first must be hydrolyzed to be metabolized in the cells. Glycosidases (EC 3.2.1) are enzymes that hydrolyze the glycosidic bonds of polysaccharides, hence facilitating the release of the smaller sugars involved in nutrient acquisition. Therefore, we have determined whether an increased glycosidase activity could augment any prebiotic character of the 2-substituted (1,3)-β-d-glucan synthesized by *P. parvulus* 2.6. Therefore, the β-glycosidase (*bgl*, lp_3629) gene from *L. plantarum* strain WCFS1, which was previously characterized in response to abiotic stress [20], was cloned into the pGIZ906 vector and overexpressed in *L. plantarum*. Overproduction of Bgl in the recombinant strain (named *L. plantarum* WCFS1β-gal) was confirmed by SDS-PAGE. Figure 1 shows that this strain produced large amounts of a 53 kDa protein, corresponding to the theoretical molecular mass of Bgl. Acebrón *et al*. [21] demonstrated biochemically that the lp_3629 gene encodes a functional β-galactosidase (EC 3.2.1.23). However, the amino acid sequence of Bgl shows that it belongs to the glycosyl hydrolase family 1. Enzymes in this family have a broad substrate specificity [22]. Therefore, the glucosidic bonds of the *P. parvulus* 2.6 β-glucan could be hydrolyzed by Bgl.

### 2.2. Prebiotic Characterization of EPS

Furthermore, we analyzed the potential prebiotic effect of EPS from bacterial origin on the survival of three strains belonging to the *Lactobacillus* genus: *L. plantarum* WCFS1, the isogenic recombinant *L. plantarum* WCFS1β-gal and *Lactobacillus acidophilus* strain NCFM. These microorganisms are known for their beneficial contribution to human health and are widely distributed in fermented food [2,23,24].

**Figure 2:**
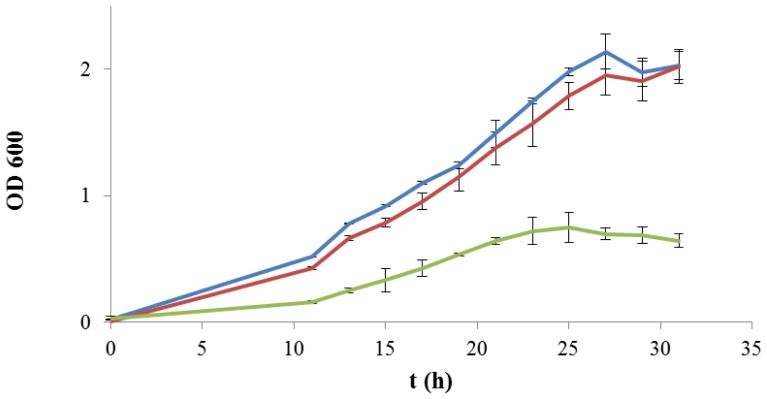
Kinetics of growth of *L. plantarum* WCFS1 (blue line), *L. plantarum* WCFS1β-gal (red line) and *L. acidophilus* (green line) in a chemically defined medium (CDM) supplemented with d-glucose (10 g·L^−1^). Growth was monitored during 30 h by spectrophotometric determination of the optical density at 600 nm (OD_600_).

The hypothetical role of the 2-substituted (1,3)-β-glucan produced by *P. parvulus* 2.6 as a sugar source for bacterial metabolism was evaluated by testing for microbial growth in a chemically defined medium which has been recently described [25]. To confirm that a source of carbohydrate was essential for bacterial development, the medium was initially prepared omitting d-glucose. Under this condition, no growth was detected for any of the strains examined (data not shown), in agreement with Terrade and Mira de Orduña [26], who did not observe biomass yield in the same medium without d-ribose as a unique sugar substrate. When only d-glucose was added to the medium as a sugar source, both *L. plantarum* WCFS1 and *L. plantarum* WCFS1β-gal strains showed a very similar growth kinetics, reaching the stationary phase after approximately 24 h (OD_600_ = 2.00) (Figure 2). In contrast, *L. acidophilus* exhibited a much lower growth at all the monitored times. The maximum cell concentration was reached after 24 h (OD_600_ = 0.75), followed by a death-phase after 30 h (OD_600_ = 0.65) (Figure 2), as even confirmed by plate count analysis (data not shown). Since the medium used in this work was optimized to analyze LAB isolated from wine [25], the reduced growth observed for *L. acidophilus* may be related to the composition of the growth media.

**Figure 3:**
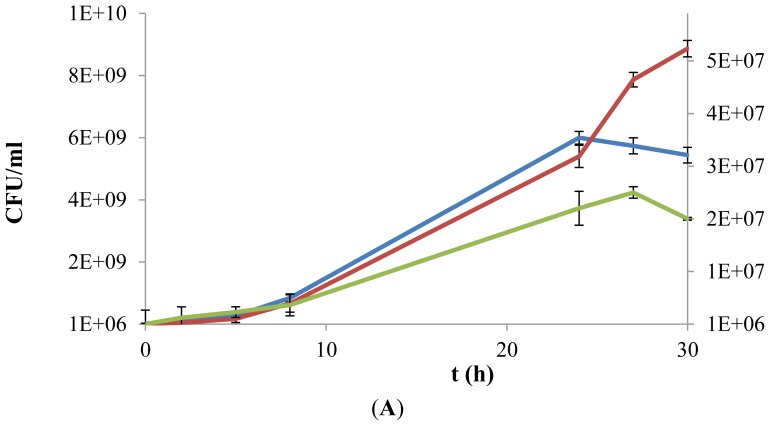

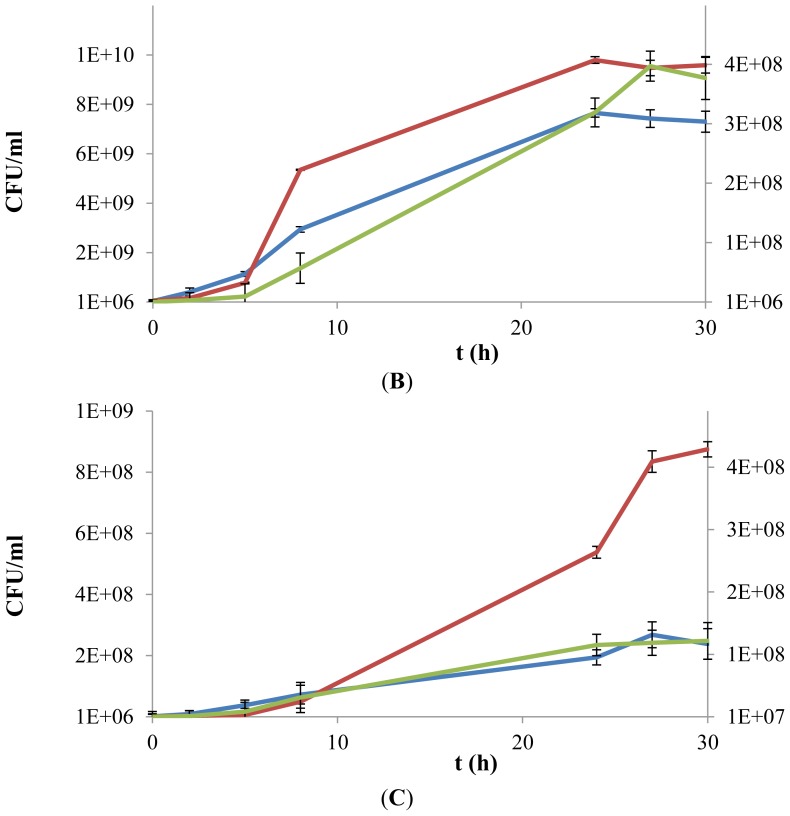
Growth kinetics of *L. plantarum* WCFS1 (**A**), *L. plantarum* WCFS1β-gal (**B**) and *L. acidophilus* (**C**) in a chemically defined medium supplemented with d-glucose (10 g L^−1^) (blue line), exopolysaccharides (EPS) (0.5% *w*/*v*) (green line) or both (red line). Values of growth in presence of only EPS are reported on a secondary *Y*-axis. Growth was monitored over a 30 h period, by colony forming units (cfu) counting.

The exopolysaccharide used in this study was obtained from *P. parvulus* 2.6 and the yield of purified EPS was about 200 mg per liter of microbial culture, as previously reported by Garai-Ibabe *et al.* [14]. Therefore, due to their low availability, the experimental assays in presence of EPS were performed in microtitre plates. The concentration of viable cells, expressed as colony forming units (cfu), was determined at incubation times 0, 3, 6, 9, 24, 27, and 30 h (Figure 3). In the *L. plantarum* strains analyzed (Figure 3A,B), a significant difference of more than 1-log was observed when bacteria were grown in a medium supplemented with only d-glucose or with EPS as carbon source. In addition, a beneficial effect was also observed in the viability of the two strains when inoculated in medium containing d-glucose, supplemented with EPS. In particular, *L. plantarum* WCFS1β-gal showed an increased biomass in all phases when both substrates (d-glucose and EPS) were in the medium, reaching a maximum cell viability of 9.8 × 10^9^ cfu mL^−1^ after 24 h, followed by a subsequent slight decrease (Figure 3B). A similar pattern of growth was observed in presence of only glucose, but a reduced viability was detected in the logarithmic and stationary phase, with a maximum concentration corresponding to 7.7 × 10^9^ cfu mL^−1^ after 24 h (Figure 3B). In contrast, *L. plantarum* WCFS1 showed an identical overlapping of the viability during the first 24 h (approximately 6.0 × 10^9^ cfu mL^−1^), independently from the presence or absence of EPS in addition to d-glucose. Interestingly, after the critical time of 24 h of incubation, bacteria where still able to grow in EPS-containing media (around 9.0 × 10^9^ cfu mL^−1^, at 30 h), while their number gradually declined in media supplemented with only d-glucose (5.4 × 10^9^ cfu mL^−1^, at 30 h) (Figure 3A).

The growth rate observed for *L. acidophilus* in the presence of only EPS was lower compared to the assay carried out when only d-glucose was provided, with plate counts of 1.3 and 2.3 × 10^8^ cfu mL^−1^, as maximum viable cell concentration, respectively (Figure 3C). Surprisingly, the availability of both substrates stimulated the growth of *L. acidophilus* by delaying its entry into stationary phase. Indeed, after 24 h the cfu were 2.5-fold more copious than in the other conditions, and at the last experimental time a value of 8.7 × 10^8^ cfu mL^−1^ was reached (Figure 3C).

The results reported in this study revealed a positive effect of the β-d-glucan on the growth of the investigated strains, suggesting that its use as a prebiotic may positively modulate the growth of probiotic organisms. In particular, the availability of both d-glucose and EPS extended the logarithmic phase for both *L. plantarum* WCFS1 and *L. acidophilus* strains. To explain this result, we assume that after the immediate consumption of d-glucose, bacteria could use EPS as a further carbon source. The release of hydrolytic enzymes under conditions of starvation, or the lysis of bacteria occurring at late exponential or stationary-phase, would enhance the activity of glycosidases in the extracellular environment. It is well known that the microbial species investigated in this study have a genetic makeup that allows them to use a variety of carbohydrate sources. Indeed, *in silico* analysis of the *L. plantarum* WCFS1 genome predicted 25 complete phosphoenolpyruvate sugar-transferase systems (PTS) enzyme II complexes, several incomplete complexes and 30 transporter systems that are involved in the transport of carbon sources [27]; likewise, the genome of *L. acidophilus* encodes a large variety of genes related to carbohydrate utilization, including 20 PTS and five transporters of the ABC family [24]. In addition, the substrate specificity cannot be predicted for some PTS and other carbon-uptake systems, and various sugar transport systems are known to import more than one substrate, thereby expanding the carbon transport capacity of these species even further [27].

In the medium supplemented with only EPS, we always observed an increase in the number of viable cells after 30 h of incubation, corresponding to approximately 1 log for *L. acidophilus* and *L. plantarum* WCFS1, while for the engineered *L. plantarum* WCFS1β-gal strain, viable bacteria numbers increased from 1.3 × 10^6^ cfu mL^−1^ to 3.8 × 10^8^ cfu mL^−1^. In addition an efficient co-metabolism of glucose and EPS was observed for this strain, which resulted in an increase of cell viability. This confirms that the overexpression of the lp_3629 gene encoding a functional β-glycosidase [21] could lead to a more marked hydrolysis of EPS, thus enhancing bacterial survival. Expression of similar enzymes might be a widespread strategy of the gut synbiotic bacteria. Indeed, in order to survive in the lower intestinal tract, bifidobacteria produce various kinds of exo- and endoglycosidases in surface-bound and/or extracellular forms, by which they can utilize diverse carbohydrates [28]. Recent genome sequence analysis of the probiotic *Bifidobacterium longum* NCC2705 revealed that more than 8.5% of the total predicted proteins were involved in the degradation of oligo- and polysaccharides, suggesting a superior ability of this organism to adapt to the poor nutritional conditions of the low intestine [29].

We also found that the growth of *L. plantarum* WCFS1 and *L. acidophilus* was favored by the simultaneous availability of both substrates, suggesting a synergistic effect of EPS and d-glucose in promoting bacterial viability. However, further studies are necessary to explain the biochemical and molecular bases of these observations.

**Figure 4:**
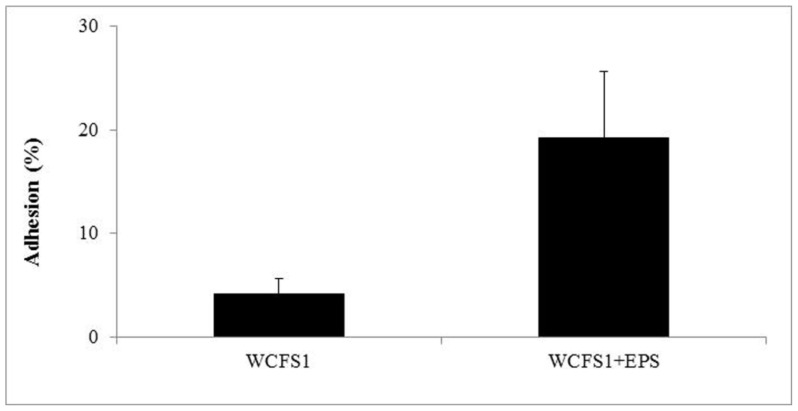
Percentage of adhesion of *L. plantarum* WCFS1 to Caco-2 cells after incubation with, or without EPS (0.5% *w*/*v*).

Because EPS are known to be involved in biofilm formation [30], several authors have investigated *in vitro* the link between EPS production and the capability to colonize the intestinal environment [13,14]. However, such studies did not contemplate the influence that exogenous EPS could have on the adhesion level of other microbial species. Therefore, in this work we investigated the effect of the 2-substituted (1-3)-β-d-glucan on the adhesive ability to human intestinal epithelial cells of *L. plantarum* WCFS1, a strain that does not show any mucous production in culture medium supernatants. In the absence of the purified EPS, approximately 4% of the bacteria adhered to Caco-2 cells. This is consistent with previous work reporting a percentage of adhesion ranging from 2% to 10% for commercial probiotic strains [31]. However, when *L. plantarum* was incubated with 0.5% *w*/*v* of EPS, the percentage of bound bacteria increased approximately five-fold (Figure 4). It has already been demonstrated that prebiotics such as oligosaccharides can influence adhesion of specific strains [32]. Intriguingly, to the best of our knowledge, no global positive effects of these molecules on microbial adhesion potential has been detected [32–35].

As components of the bacterial cell surface, the EPS produced and secreted by LAB are likely to underlie inter-cellular recognition mechanisms and/or the process of biofilm formation. In this regard, β-d-glucans might promote the initial steps of adhesion to host intestinal cells. This finding raises considerable interest for its potential applications in the design of symbiotic food products. Indeed, we can speculate that addition of specific EPS in the food matrix that vehicles probiotics might enhance microbial adhesion to the intestinal epithelium, thus improving gut colonization by beneficial microorganisms. A persistent or at least transient colonization of the gut is a desirable feature of probiotics, as a close and prolonged interaction with the host allows them to accomplish beneficial effects such as immune function modulation and pathogen exclusion [36]. Moreover, as observed for other prebiotic NDO, β-d-glucans might also contrast pathogen adhesion to the intestinal mucosa and/or act as antioxidants to protect probiotics from the intestinal hostile environment [37]. Further research shall be undertaken to address these issues and test such hypothesis.

## 3. Experimental Section

### 3.1. Bacterial Strains and Media

The bacterial strains used in this study were *L. plantarum* strain WCFS1 [27], its recombinant isogenic strain that overexpresses the *bgl* gene encoding a β-galactosidase (*L. plantarum* WCFS1β-gal) and the *L. acidophilus* strain NCFM [24,38].

A chemically defined medium was prepared as reported by Terrade *et al*. [25] but without d-ribose and modifying the final pH at 6.2. The growth kinetics was evaluated either in absence of any carbon source or supplementing the same medium with d-glucose (10 g·L^−1^), EPS (0.05% *w*/*v*) or both. The recombinant strain was grown in media that included 10 μg·mL^−1^ of erythromycin. *L. plantarum* strains and *L. acidophilus* were always incubated without shaking at the optimal temperature of 30 °C and 37 °C, respectively.

*P. parvulus* 2.6 [9] was isolated from ropy cider at the Department of Applied Chemistry, Faculty of Chemistry (University of the Basque Country UPV/EHU, San Sebastián, Spain).

Strains were kept in de Man Rogosa Sharpe (MRS) broth (Pronadisa, Madrid, Spain) supplemented with 20% (*v*/*v*) glycerol for long-term storage at −80 °C.

### 3.2. Production of EPS by *P. parvulus* 2.6

To obtain a pre-inocula for EPS production, *P. parvulus* 2.6 was grown on MRS broth, supplemented with 0.05% (*w/v*) L-cysteine hydrochloride (Merck, Darmstad, Germany) and 0.1% (*w*/*v*) Tween 80 (Pronadisa, Barcelona, Spain). For production of the EPS, *P. parvulus* 2.6 was grown in the semi-defined media MST [39], containing glucose (50 g·L^−1^) and ethanol 4.9% (*w*/*v*), in a 3-L fermenter (Bioflo 110, New Brunswick Scientific, Enfield, CT), at 30 °C for 96 h. The pH was controlled at 5.2 with 5 N NaOH, the agitation was kept at 50–70 rpm to keep the fermentation broth homogeneous, and nitrogen gas (0.2 L·h^−1^) was sparged through the headspace continuously to maintain anaerobic conditions.

Bacterial cells were removed from fermented media by centrifugation (16,000× *g*, 4 °C, 30 min) after 96 h of culture. The clear supernatant was collected and the EPS precipitated by adding three volumes of cold absolute ethanol, and maintained overnight at 4 °C. The precipitate was recovered by centrifugation at 14,000× *g* for 10 min at 4 °C, the resulting EPS pellet was dissolved in deionized water, and the EPS was recovered by three cycles of precipitation with ethanol. The final precipitate was dissolved in and dialyzed against deionized water, using a dialysis membrane (Medicell International, Ltd., London, U.K.) having a cut-off of 3.5 kDa, for 2–3 days (water changed twice each day). After dialysis, the precipitate was lyophilized.

For the experimental assay, media were prepared by adding appropriate volumes of a 100× concentrated stock solution of EPS to obtain the desired final concentration.

### 3.3. Overexpression of Bgl Gene in *L. plantarum*

All the plasmid constructions were performed in *Escherichia coli* TG1 strain. The *bgl* gene was cloned in the pGIZ906 vector, a pLAB1301-derivative with a 0.352-kb insert containing the *ldhL* gene expression signals of *L. plantarum* WCFS1 [27,40]. The *bgl* gene was amplified from *L. plantarum* WCFS1 genomic DNA using forward (5′-AAAACTGCAGGAGTTCCGGAAGGCTTT-3′) and reverse (5′-GCTCTAGATCAAAACCCATTCCGTTCCCCA-3′) primers, harbouring *Psti*I and *Xba*I sites (underlined sequence), respectively. The PCR fragment was digested with *Pst*I–*Xba*I and located between the *Nsi*I–*Xba*I sites of pGIZ906 vector by ligation to generate a PldhL-bgl transcriptional fusion. The recombinant plasmid was electroporated into *L. plantarum* WCFS1 and transformants were identified on MRS plates containing erythromycin. Plasmid DNA preparation was performed following standard procedures. The absence of mutations in the plasmidic *bgl* gene was confirmed by DNA sequencing.

### 3.4. Protein Extraction and SDS-PAGE

Cells of *L. plantarum* β-gal grown in 30 mL of MRS to middle exponential phase were sedimented by centrifugation (5000× *g*, 10 min, 4 °C) and washed twice with 0.1 M sodium phosphate buffer pH 7.0. Bacteria were resuspended in 600 μL of cold phosphate buffer containing 0.1 mM phenylmethylsulfonyl fluoride (PMSF) (Roche Applied Science, Indianapolis, IN), mixed with glass beads (212–300 μm Ø, Biospec Products, Bartlesville, OK) and mechanically disrupted in a Mini Beadbeater™ (Biospec Products) (four cycles of 1 min at max speed followed by 1 min on ice). After removal of cellular debris by centrifugation (13,000× *g*, 10 min, 4 °C), supernatants were aliquoted and stored at −20 °C. Quantification of total protein extracts was determined with a Bio-Rad Protein Assay (Biorad, Milan, Italy), according to the manufacturer’s instructions. Protein fractionation was carried out by sodium dodecylsulphate-polyacrylamide gel electrophoresis (SDS-PAGE) at 150 V for 1 h in a 12% (*w*/*v*) resolving and 4 % (*w*/*v*) stacking gel. A prestained SDS-PAGE standards, low range (Biorad) was used as protein marker. After PAGE, protein bands were revealed by staining with coomassie brilliant blue (0.2% CBB R-250).

### 3.5. Kinetics of Cell Growth with Different Carbon Sources

Strains from glycerol stocks were inoculated into MRS medium and grown to middle exponential phase (OD_600_ = 0.8 and for *L. plantarum* and OD_600_ = 0.6 for *L. acidophilus* strains). These cultures were used as inocula (dilution 1:100 in fresh defined medium), after harvest by centrifugation (5000× *g*, 5 min), two washes and resuspension in the corresponding chemically defined medium. The kinetics of bacterial growth were spectrophotometrically monitored during 24 h in media supplemented or not with d-glucose. The analysis was performed in the wells of microtitre plates containing 200 μL of chemically defined medium in the presence of only glucose, only EPS or both. The growth of all strains was analyzed by determination of colony forming units (cfu) by plate count of samples incubated for 30 h at optimal temperature. Experiments were performed in triplicate.

### 3.6. Adhesion Assay

Adhesion assays were performed using the Caco-2 epithelial cell line, originated from human colonic carcinoma, and kindly gifted by Dr. C. Lamacchia, University of Foggia. Caco-2 cells were grown as previously described [14,31,41] and supplemented with 2 mM l-glutamine (Sigma-Aldrich, St. Louis, MO). In post-confluent cultures, the viable cell number, as counted in a Burker chamber, was about 4.3 × 10^4^ cells per well and bacteria were added to achieve a final concentration of approximately 4.3 × 10^8^ cfu/mL (ratio 1000:1 bacteria to Caco-2 cells). Adhesion experiments were then performed according to Bove *et al*. [41]. Serial dilutions of the samples were plated onto MRS-agar plates to determine the number of cell-associated bacteria (viable counts) expressed as cfu. The adhesion percentage was calculated by comparing the number of cfu from washed wells (cell-bound bacteria) with those from control unwashed wells (unbound and bound bacteria). Experiments were performed in triplicate.

### 3.7. Statiscal Analysis

Significant differences between averages of duplicate measurements were evaluated by performing *t*-tests after analysis of variance at a confidence level of *p* = 0.05.

## 4. Conclusions

In this work we reported the prebiotic action of a 2-substituted (1-3)-β-d-glucan of bacterial origin on three probiotic LAB. In particular, our results showed that all the investigated bacterial strains were able to use EPS as a substrate for their growth. In addition, the simultaneous availability of d-glucose and EPS as glucid sources delayed the entry into stationary phase of *L. plantarum* WCFS1β-gal and *L. acidophilus*, as well as encouraged the growth of *L. plantarum* WCFS1 and *L. acidophilus* in the exponential-phase, thus suggesting their synergistic effect in promoting metabolic processes. The pronounced rate of growth observed for the recombinant strain *L. plantarum* WCFS1β-gal confirms the positive influence of glycosidases enzymes in increasing the prebiotic potential of EPS. Notably, this β-d-glucan increases the binding of the non-ropy *L. plantarum* WCFS1 strain to intestinal cells. These results suggest that mixed probiotic LAB strains, able either to produce or consume β-glucans, could be a suitable strategy for the development of new types of functional food.
